# Activity in GEriatric acute CARe (AGECAR): rationale, design and methods

**DOI:** 10.1186/1471-2318-12-28

**Published:** 2012-06-09

**Authors:** Steven J Fleck, Natalia Bustamante-Ara, Javier Ortiz, María-Teresa Vidán, Alejandro Lucia, José A Serra-Rexach

**Affiliations:** 1 Sport Science Department, Colorado College, Colorado Springs, CO, USA; 2 Universidad Europea de Madrid, 28670 Villaviciosa de Odón, Madrid, Spain; 3 Geriatrics Department, Hospital General Universitario Gregorio Marañón, Madrid, Spain

**Keywords:** Randomised controlled trial, Ageing, Hospitalisation, Elderly, Intrahospital exercise, Functional capacity

## Abstract

**Background:**

The Activity in GEriatric acute CARe (AGECAR) is a randomised control trial to assess the effectiveness of an intrahospital strength and walk program during short hospital stays for improving functional capacity of patients aged 75 years or older.

**Methods/Design:**

Patients aged 75 years or older admitted for a short hospital stay (≤14 days) will be randomly assigned to either a usual care (control) group or an intervention (training) group. Participants allocated in the usual care group will receive normal hospital care and participants allocated in the intervention group will perform multiple sessions per day of lower limb strength training (standing from a seated position) and walking (10 min bouts) while hospitalized. The primary outcome to be assessed pre and post of the hospital stay will be functional capacity, using the Short Physical Performance Battery (SPPB), and time to walk 10 meters. Besides length of hospitalization, the secondary outcomes that will also be assessed at hospital admission and discharge will be pulmonary ventilation (forced expiratory volume in one second, FEV_1_) and peripheral oxygen saturation. The secondary outcomes that will be assessed by telephone interview three months after discharge will be mortality, number of falls since discharge, and ability to cope with activities of daily living (ADLs, using the Katz ADL score and Barthel ADL index).

**Discussion:**

Results will help to better understand the potential of regular physical activity during a short hospital stay for improving functional capacity in old patients. The increase in life expectancy has resulted in a large segment of the population being over 75 years of age and an increase in hospitalization of this same age group. This calls attention to health care systems and public health policymakers to focus on promoting methods to improve the functional capacity of this population.

**Trial registration:**

ClinicalTrials.gov ID: NCT01374893.

## Background

Due to Western societies populations living longer there is a demand to explore new ways to promote healthy ageing instead of merely treating the diseases of old age [1]. According to the United Nations (average for the 2005–2010 period), Spain has the sixth longest life expectancy at birth in the world [2]. Therefore, especially in Spain, it is of public health and clinical relevance to better understand the effects of regular physical activity in old people. This is not only true for non-hospitalized individuals, but also includes hospitalized older individuals.

The negative effect of hospitalisation on functional outcomes in population-based [3] and in-hospital cohort studies is well-established [4,5]. This negative effect occurs even with short (several days) hospital stays [6-8]. Ten days of bed rest result in significant losses of whole body lean tissue, lower body lean tissue and strength in healthy 67-year-olds [7]. Loss of strength with bed rest can be as great as 5% per day [9]. With such significant losses of strength it is not surprising that even short hospital stays result in a decrease in functional capacity, including the ability to cope with activities of daily living (ADLs). In fact 30–50% of seniors admitted for a short hospital stay show a decrease in functional capacity at discharge [10-14]. These decreases in functional capacity are associated with an increased risk of mortality. For example, individuals showing the least decrease in functional capacity with a hospital stay have a mortality rate of 10.7% three months after discharge compared to 36.7% in individuals showing the greatest decrease in functional capacity [15].

As much as 73% and 83% of the measured hospital stay of older individuals is spent lying in bed [16,17]. During short hospital stays complete bed rest and low mobility levels are associated with negative functional outcomes [6,8]. Compared to patients with higher mobility levels, patients (mean age of ~79 years) who had complete bed rest or low mobility levels of all types (physical therapy, activity initiated by others, self initiated activity) during short hospital stays (5–8 days on average) showed greater declines in the ability to cope with ADLs, and greater risk of new institutionalization and death upon discharge and at 30 days after discharge [6,8]. The relationship between low mobility levels and negative functional outcomes is still significant after statistical multivariable adjustment for co-morbidity factors, such as severity of illness [6]. In contrast, the decline in negative outcomes with higher mobility levels follows a dose–response effect [8].

A recent meta-analysis concluded that intrahospital programs with the goal of maintaining or increasing functional capacity in hospitalized seniors result in decreased mortality rates, decreased length of hospitalization and decreased rates of discharge to nursing homes compared to discharge to home [18]. This indicates mobility and physical training are modifiable factors that have important implications for care regimes. Loss of lower body muscle mass is a strong predictor of physical performance and functional capacity in seniors [19], indicating physical training programs should emphasize the lower body musculature. Therefore, it is important to determine if increased mobility and physical training, especially of the lower body, have an effect upon functional outcomes during short hospital stays in older individuals.

### Objectives

The purpose of the proposed research [acronym: AGECAR (Activity in GEriatric Acute CARe)] is to investigate the effect of an intrahospital multi-time per day physical training program, consisting of walking and strength training of the lower limbs, during a short period of hospitalization in seniors aged 75 years or older on functional capacity at discharge (primary outcome). The effect of the physical training program will be compared to a control group receiving normal hospital care. Besides length of hospitalization, the secondary outcomes that will also be assessed at hospital admission and discharge will be pulmonary ventilation (forced expiratory volume in one second, FEV_1_) and peripheral oxygen saturation. The secondary outcomes that will be assessed by telephone interview three months after discharge will be mortality, number of falls after discharge, and ability to cope with activities of daily living (ADLs, using the Katz ADL score and Barthel ADL index).

Increased age and a history of falls in the previous year are associated with decreased functional capacity [11], and handgrip strength is a predictor of survival [20] and mobility [21]. On the other hand, low cognitive ability, reflected as low scores on the Mini-Mental Status Examination (MMSE) is associated with increased chance of decreased functional capacity upon discharge [11,22]. Thus, we will also determine if age, history of falls in the previous year, handgrip strength and MMSE scores (covariates) will have an influence on the effects of the training program on the aforementioned primary and secondary outcomes. The major hypothesis is the prescribed training program will increase functional capacity at discharge while decreasing length of hospitalization.

## Methods/Design

### Study design

**Figure 1:**
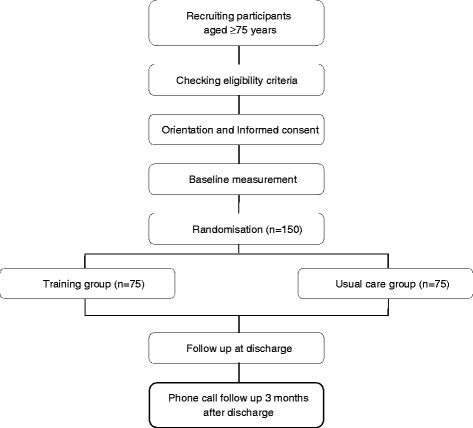
Study flow diagram.

The present study is a randomised controlled trial (RCT) (ClinicalTrials.gov ID: NCT01374893) and is designed to be compliant with the recommendations of the Consolidated Standards of Reporting Trials (CONSORT) statement [23]. The study flow diagram is shown in Figure 1. After signing an informed consent form subjects will be randomly assigned (as explained below) to either the intervention or control group. The intervention group will perform the multi-session per day training program while the control group will receive normal hospital care. Due to the possible effect of subjects in the control group seeing subjects in the intervention group performing physical activity in addition to normal hospital care, randomization will take place in a time dependent manner. Patients admitted during four-week blocks of time will all be assigned to either the treatment or control group, with a one week period between four-week periods. The participants will be followed for the length of their hospital stay, and also with a follow-up phone interview three months after discharge to determine mortality rate at three months, number of falls since discharge, and to subjectively assess the ability to cope with ADLs using the Katz ADL score [24] and the Barthel ADL index [25].

All testing (at admission and discharge) will be performed in the same setting (*Hospital General Universitario Gregorio Marañón*, Madrid, Spain) and by the same investigators. The study will be performed between June 2012 and June 2013, following the ethical guidelines of the Declaration of Helsinki, last modified in 2000.

### Study participants and selection criteria

Participants will include approximately 150 elderly people aged 75 years or older recruited from patients admitted into the Geriatrics Department of the *Hospital General Universitario Gregorio Marañón* (Madrid, Spain). Due to the length of the study it is possible a patient will be readmitted after participating in either the experimental or control group. To avoid this confounding variable, a study participant who is readmitted to the hospital during the course of the study will not be included in the study population a second time.

The inclusion criteria are:

Age: 75 years or over.

Able to ambulate, with or without personal/technical assistance.

Able to communicate.

Informed consent: Must be capable and willing to provide consent.

The exclusion criteria are:

Duration of hospitalization < 72 hours

Any factor precluding performance of the physical training program or testing procedures as determined by the attending physician. These factors include, but are not limited to the following:

Terminal illness.

Myocardial infarction in the past 3 months.

Not capable of ambulation.

Unstable cardiovascular disease or other medical condition.

Upper or lower extremity fracture in the past 3 months.

Severe dementia.

Unwillingness to either complete the study requirements or to be randomised into control or intervention group.

Presence of neuromuscular disease or drugs affecting neuromuscular function.

### Randomisation and blinding

As mentioned above, randomization will be done in a time-dependent manner (in four-week blocks) in order to avoid the confounding variable of participants in the control group seeing subjects in the treatment group performing physical activity in addition to normal hospital care. Participants will be explicitly informed and reminded not to discuss their randomisation assignment with assessment staff. The assessment staff will be blinded to participant randomisation assignment, as well as to the main study design and to what changes we expect to occur in the study outcomes in either group. Because blinding of assessment staff to participant randomisation assignment might not be actually feasible in all cases due to the time-dependent manner of randomisation, we will record blinding success and add it as a covariate in the analyses (see below). It will not be possible to conceal the group assignment from the staff involved in the training of the intervention group.

### Sample size and statistical power

The required sample size was determined for one of the primary outcome variables, i.e. the SPPB. We believe that a clinically relevant change is a ≥30% increase in the aforementioned test battery. We expect the control group to improve ~0 to 5%; thus, we can detect differences of at least ≥35% with a power >80% and a level of significance of 0.05 with two groups of 60 subjects. Assuming a maximum loss of follow-up of 20%, we will recruit a total of 150 patients.

### Usual care group (control)

Participants randomly assigned to the usual care group will receive normal hospital care, which includes little physical activity, i.e. short daily walk in all patients at risk for delirium or functional decline.

### Intervention (training)

The intervention will consist of strength training of the lower body and walking. Both types of training will be performed three times per day during the week (Monday to Friday), two times per day on Saturday, beginning as soon as possible after admission and continuing until discharge. No training will take place on Sunday. Training sessions will be dispersed throughout the day. When three sessions per day are performed, one will take place between 9–11 a.m., one between 13–15 p.m., and the last one between 17–19 p.m.; when two sessions per day are performed training will take place during the first two of the aforementioned time periods. Strength training will be performed first, and followed by walking. A rest period of up to five minutes will be allowed between the strength and walk training.

Strength training will consist of rising from a seated position in a chair to an upright position with or without the use of the subject’s hands on the armrests of the chair. Initially 10 repetitions or as many repetitions as possible up to 10 of rising from a seated position will be performed each training bout. The strength training will be progressed from one set of the 10 repetitions to 2 and then 3 sets of the 10 repetitions based on individual progress. When a patient can perform one set of 10 repetitions for all three training bouts on two consecutive days a second set will be added. When a patient can perform two sets of 10 repetitions for all three training bouts on two consecutive days a third set will be added.

Completion of one set of 10 repetitions of strength training should take approximately one-two minutes. A two-minute rest period will be allowed between strength training sets if more than one set is performed. If a subject completes three sets of strength training total training time will be between 7 and 10 minutes.

Walk training will consist of walking as far as possible with or without assistance for 10 minutes. Total length of each training session will be 20–23 minutes. During the week when three training sessions per day are performed total training time per day will be 60–69 minutes. On Saturdays when two training sessions per day are performed total training time will be 40–46 minutes. All training sessions will be individually monitored with a record of training completed compiled.

### Participant retention and adherence

To reduce participant drop out and to maintain adherence to the training program, the potential benefits of performing the training will be explained to subjects in the intervention group. Qualified fitness specialists will individually monitor and carefully supervise all training sessions and provide instruction and encouragement during all training sessions. Distribution of the training sessions throughout the day should minimize cumulative fatigue and so help maintain adherence. Adherence to the exercise intervention program will be checked in a daily register of sessions.

### Demographics

A standard questionnaire including name, residence, age, reason for hospitalisation, medical history, current dosage of medications being taken and history of falls in the last year will be administered by trained investigators.

### Primary outcome (functional capacity) measures

The Short Physical Performance Battery (SPPB) will be used to assess participants’ physical ability at baseline (admission) and discharge. The SPPB consists of performance of three different tests (repeated chair stand test, time needed to walk a distance of 4 meters two times, and a hierarchical standing balance test), with the scores on all three tests combined resulting in a composite, continuous score of 0 to 12, as explained below [26]. The repeated chair stand test consists of rising from and sitting in a chair five consecutive times whereas the walking test consists of walking 4 meters two times with or without the assistance of a cane or walker. Scoring in each of the aforementioned two tests is done on a 0–4 point scale, where those who cannot complete the task are assigned a score of 0 and those completing the task are assigned a score of 1 to 4, corresponding to the quartiles of time needed to complete the task, with the fastest times scored as 4 [26]. For the hierarchical standing balance task, participants are first asked to place their feet in a side-by-side position, followed by a semitandem position (heel of one foot along side the big toe of the other foot) and tandem position (heel of one foot directly in front of the other foot) [27]. Participants are required to hold the side-by-side position for 10 seconds to advance to the semitandem task, and to advance to the tandem task the semitandem position must be held for 10 seconds. Those who cannot hold the side-by-side position for 10 seconds are assigned a score of 0 whereas those who can hold it for 10 seconds but are unsuccessful in holding the semitandem position for 10 seconds receive a score of 1. A score of 2 is given if the semitandem is held for 10 seconds but the tandem cannot not be held for more than 2 seconds, a score of 3 is given if the tandem is held for 3–9 seconds, and a score of 4 if the tandem is held for 10 seconds. The total score for the SPPB is the sum of the score on all three tests, i.e. in a continuous 0–12 point scale.

Time to walk 10 meters will be also determined as a test of the participants’ functional capacity at baseline (admission) and discharge. The participant will be asked to ambulate 10 meters as quickly as possible with or without assistance from a cane or walker.

All SPPB and 10-meter walk tests will be administered by the same investigator with time determined to the nearest 0.1 second.

### Secondary outcome measures

#### *Length of hospitalization*

We will assess length of hospitalization (in days).

#### *Pulmonary ventilation*

Pulmonary ventilation is negatively affected by aging due to decreased ability to expand the rib cage resulting in an increased lung residual volume [28]. The inability to expand the rib cage is exasperated with bed rest, which can eventually affect the ability of the pulmonary system to exchange oxygen and carbon dioxide [29,30]. Pulmonary ventilation will be assessed at admission and discharge as forced expiratory volume in one second (FEV_1_). To measure FEV_1_ the patient will be asked to inhale maximally and then exhale maximally while in a seated position into a hand held flow meter (Flow Screen, Jaeger Company, Viasys Healthcare GmbH, Germany).

#### *Peripheral oxygen saturation*

Peripheral oxygen saturation (SpO_2_) decreases from approximately 85 to 77 mmHg in seniors when moving from a standing to a supine position [31]. Such decreases can result in symptoms of confusion in patients already at the threshold of pulmonary insufficiency [28]. Physical activity also results in a decrease in partial pressure of oxygen. SpO_2_ will be determined at admission and discharge, immediately pre and immediately post of the 10-meter walk test using a finger pulse oximeter (Rossmax Inno Tek Corp, Taiwan).

#### *Assessment after discharge*

We will perform a follow-up phone interview with participants (or their relatives/caregivers in case of death) three months after discharge, to determine mortality rate after three months and number of falls within three months after discharge, and also to subjectively assess the ability to cope with ADLs using the Katz ADL score [24] and the Barthel ADL index [25].

The Katz ADL scale includes six items (eating, transferring from bed to chair, walking, using the toilet, bathing, and dressing) each of which is scored with 0 (= unable to perform the activity without complete help), 0.5 or 1.0 (= able to perform the activity with little help or without any help, respectively) [24]. A sum-score (ranging from 0 to 6) is given for each patient. The Barthel index is an instrument widely used to measure the capacity of a person for the execution of ten basic activities in daily life, obtaining a quantitative estimation of the subject’s level of independency [25,32]. The ten items include: eating, transferring from bed to chair, using the toilet, bathing/showering, personal hygiene (e.g., tooth brushing, shaving) dressing, walking, stair climbing, and bowel and bladder control. Each individual item is scored with 0 (i.e. unable to perform without complete help or fecal/urine incontinence), 5 (i.e. able to perform the activity with little help or only accidental fecal/urine incontinence) or 10 (i.e. able to perform without any help or total fecal/urine continency). The sum-score ranges from 0 (*totally dependent*) to 100 (*totally independent*).

### Covariates

Besides actual blinding success of assessment staff to participant randomisation assignment, the following covariates will be measured in all participants upon hospital admission as they could influence one or more of the study outcomes.

#### *Body mass index*

Standing height will be measured to the nearest 0.1 cm with a clinical stadiometer (Asimed T2, Barcelona, Spain) while the person is standing barefoot. Body mass will be determined to the nearest 0.05 kg using a balance scale (Ano Sayol S.L., Barcelona, Spain) with the person in her/his underwear. Body mass index (BMI) will be calculated as weight/height (kg/m^2^).

#### *History of falls within the previous year*

We will record the participants’ history of falls within the 12-month period prior to hospital admission. A fall will be defined as an “unexpected event in which the participants come to rest on the ground, floor, or other lower level” [33,34].

#### *Maximal handgrip strength*

Handgrip strength will be measured using a digital dynamometer (T.K.K. 5101 Grip-D; Takey, Tokyo, Japan), with the scores recorded to the nearest 0.1 kg. When performing the measurement, participants will be instructed to maintain the standard bipedal position during the entire test with the arm at their side in complete extension. The dynamometer will not be allowed to touch any part of the body except the hand being tested. Each subject will perform (alternately with both hands) the test twice with a 30–60 second rest period between the measurements. For each measure, the hand to be tested first will be chosen randomly. The grip span of the dynamometer will be adjusted to the individual’s hand size [35].

#### *Cognitive ability*

The Mini-Mental State Examination (MMSE) will be administered to determine participants’ cognitive ability [36].

### Familiarization and reliability assessment

Before the start of the study all subjects will have a familiarization session consisting of an explanation of all tests and performing all tests. This session will last ~30 minutes. Test-retest reliability for each outcome measure will be determined on a sub-population of the subjects.

### Assessment of side effects

Adverse events, including muscle pain, fatigue, and general aches and pains will be recorded by the training and testing staff; and by self-report during the study period. We will also record the falls during the study. An independent researcher will be in charge of auditing all nursing and medical records to record all falls in the participants during the study period.

### Statistical analysis

To assess the training effects on the study outcomes, we will analyze the data according to the intention-to-treat principle [37]. When post-test data are missing, baseline scores will be considered post-test scores. We will use a two-factor (group and time) analysis of variance (ANOVA) with repeated measures. We will repeat the analysis using the actual blinding of assessment staff to participant randomisation assignment (successful or not), baseline values of age, body mass index, MMSE, number of falls within the previous year and handgrip strength as covariates. For each outcome variable we will report the level of significance corresponding to the main group (between-subjects), time (within-subjects) and interaction (group × time) effects. We will perform post hoc pre- vs. post comparisons by group only when a significant group*time effect is present. The level of significance will be set to = 0.05. We will adjust multiple comparisons for mass significance [38]. We will use the Student’s *t* test (or its non-parametric equivalent, the Mann Whitney’s *U* test) for comparing between the two groups the mean values of those secondary outcomes that will be assessed only once, i.e. three months after discharge (number of falls, Katz score and Barthel index). We will compare mortality rate at three months after discharge in the two groups with the chi square test.

## Discussion

The increase in life expectancy has resulted in a large segment of the population in industrialized countries being over 75 years of age. As the population ages the number of individuals over 75 years of age who are hospitalized and are not able to live independently, will likely increase. This increase in the older segment of the population and the number of older individuals hospitalized calls attention to health care systems and public health policymakers to focus on promoting methods to improve functional capacity of this sector of the population. The present study examines the feasibility and effect of an intra-hospital physical training program during brief hospital stays. Results from the current study will help to better understand the potential of this type of physical training for improving the well being of older individuals.

## Abbreviations

ADLs: activities of daily living; ANOVA: analysis of variance; BMI: body mass index; FEV1: forced expiratory volume in one second; MMSE: Mini-Mental State Examination; QOL: quality of life; RCT: randomised controlled trial; SaO2: peripheral oxygen saturation; SPPB: Short Physical Performance Battery.

## Competing interests

The authors have no competing interests.

## Authors’ contributions

SJF: 1) has made substantial contributions to conception and design; 2) has been involved in drafting the manuscript; and 3) has given final approval of the version to be published. NB-A: 1) has made substantial contributions to conception and design; 2) has been involved in revising the manuscript critically for important intellectual content; and 3) has given final approval of the version to be published. JO: 1) has made substantial contributions to conception and design; 2) has been in revising the manuscript critically for important intellectual content; and 3) has given final approval of the version to be published. M-TV: 1) has made substantial contributions to conception and design; 2) has been involved in revising the manuscript critically for important intellectual content; and 3) has given final approval of the version to be published. AL: 1) has made substantial contributions to conception and design; 2) has been involved in drafting the manuscript; and 3) has given final approval of the version to be published. JAS-R: 1) has made substantial contributions to conception and design; 2) has been involved in revising the manuscript critically for important intellectual content; and 3) have given final approval of the version to be published. All authors’ read and approved the final manuscript.

## Pre-publication history

The pre-publication history for this paper can be accessed here:

http://www.biomedcentral.com/1471-2318/12/28/prepub
